# Timing of Tympanostomy Tube Placement and Efficacy of Palatoplasty Technique on the Resolution of Chronic Otitis Media: A Cross-sectional Analysis

**Published:** 2015-07-27

**Authors:** M. S. Brgoch, K. M. Dodson, T. C. Kim, D. M. Kim, R. Trivelpiece, J. L. Rhodes

**Affiliations:** Virginia Commonwealth University, Richmond

**Keywords:** tympanostomy tube, palatoplasty, chronic otitis media, intravelar veloplasty, Furlow double z-plasty

## Abstract

**Background:** Chronic otitis media with effusion is a persistent complication essentially universal in children with cleft palate. The prevalence of chronic otitis media with effusion is hypothesized to be a result of Eustachian tube dysfunction secondary to the anomalous insertion of the palatal musculature. This study was designed to evaluate the timing of tympanostomy tube placement and the effect of primary palatoplasty technique on the recovery of Eustachian tube function and resolution of chronic otitis media with effusion. **Methods:** We performed a retrospective, cross-sectional analysis of the previous 99 consecutive patients who underwent a palatoplasty at our institution. Variables included timing of initial tympanostomy tube placement, palatoplasty technique, cleft type, and gender. These were then evaluated to assess their impact on the resolution of chronic otitis media with effusion. Resolution was established as an inverse function of the number of tympanostomy tubes placed in correlation with available audiometric/tympanographic data. For all models, a generalized linear mixed model was applied using a Poisson distribution and a log-link function where the outcome variable was the total number of tympanostomy tubes. For all tests, a *P* = .05 level of significance was used. **Results:** Of 99 palatoplasties performed, 94 patients were included in the study. Ninety-one percent of patients had documented chronic otitis media with effusion at the time of palatoplasty. Forty-four percent underwent straight-line repair with aggressive intravelar veloplasty, 36% had Furlow double z-plasty, 20% had straight-line repair without intravelar veloplasty. There was a statistically significant difference (*F*_2,83_ = 5.36, *P* = .0065) between the 3 types of repair. The mean number of tubes placed was 0.6000 ± 0.1225, 0.8519 ± 0.1776, and 1.4737 ± 0.2785 for intravelar veloplasty, Furlow double z-plasty, and straight line without intravelar veloplasty, respectively . With regard to the timing of tympanostomy tube placement, there was a trend toward statistical significant (*F*_2,83_ = 3.02, *P* = .0540) in the mean number of tube insertions was 1.4286 ± 0.4518, 0.6964 ± 0.1115, and 1.1304 ± 0.2217 when the initial set was placed before palatoplasty, at the time of palatoplasty, and after palatoplasty, respectively. **Conclusions:** Despite its inherent limitations, this study suggests that palatal musculature reconstruction via intravelar veloplasty or reorientation via Furlow double z-plasty may improve Eustachian tube function and lower the need for tympanostomy tubes in this population. In comparison with other time points, patients who underwent initial tympanostomy tube placement at the time of palatoplasty trended toward improved chronic otitis media with effusion.

Orofacial clefting is the most common craniofacial malformation in the United States and is associated with feeding, speech, facial growth, and otologic complications.^1^^,^^2^ Management of children affected by orofacial clefting requires multidisciplinary team care to provide the best outcome.^3^^-^5 Chronic otitis media with effusion (COME) is a known and persistent complication that is essentially universal in children with cleft palate.^6^^-^9 This congruency is thought to be due to Eustachian tube dysfunction secondary to the anomalous insertion of the palatal musculature into the hard palate or cleft margin.^7^^,^^10^^,^^11^ This is in contrast to the standard anatomic makeup in which the levator musculature reaches the midline to create a functional muscular sling.^7^^,^^10^^,^^12^ As a result of this aberrant musculature, poor muscle-assisted Eustachian tube dilation or subsequent constriction is commonplace.^10^

While numerous techniques have sought to reverse Eustachian dysfunction through reconstruction or reorientation of the palatal musculature, a high incidence of COME after repair has continued to be reported.^9^^,^^13^^,^^14^ Despite documentation of otologic complications and long-term sequelae, controversy remains on the most advantageous approach to managing COME in this population. Existing data support both an aggressive approach with advanced placement of ventilator tubes at the time of palatal repair and a conservative approach with ventilator tubes placed after several months of documented OME following repair.^9^,^15^^-^21 At various institutes, policy dictates tympanostomy tube placement only after being deemed necessary through continued otolaryngologic examinations with tubes not routinely placed at the time of palatoplasty.^13^ Failure to reach a consensus on tube placement protocols may be due to inconsistent study designs, confounding factors, and continually small study populations. The purpose of this study was to examine the effect of distinct palatal repair techniques and the efficacy of early tympanostomy tube placement on the recovery of Eustachian tube function and potential resolution of COME in children with cleft palate at our institution.

## METHODS

We performed a retrospective, cross-sectional analysis of the previous 99 palatoplasties performed at our institution. Patients and procedures were initially identified through the institution's electronic medical record system by using designated *Current Procedural Terminology* code 42200, indicating a primary palatoplasty. After institutional review board approval, operative reports, outpatient records, and audiologic data were collected. Craniofacial and otologic variables examined included timing of initial tympanostomy tube placement (before lip repair, at lip repair, at cleft repair, or after cleft repair), cleft type, gender, palatoplasty technique, audiometry data, and tympanograms. These were then evaluated to assess their impact on the resolution of COME.

Resolution of OME was established as an inverse function of the number of tympanostomy tubes placed in correlation with available audiometric/tympanographic data. Resolution was then verified using available otolaryngologic examinations and adjunct data. For all models, a generalized linear mixed model was applied using a Poisson distribution and a log-link function where the outcome variable was the total number of tympanostomy tubes. A *P* value of .05 was used as the cutoff for statistical significance.

## RESULTS

Of 99 primary palatoplasties performed, 95 procedures were primary palatoplasties and a total of 94 patients were included in the study. Exclusion criteria consisted only of inadequate medical records for analysis. Of the 94 patients included in the study, 91.0% had documented OME at the time of palatoplasty. Gender and cleft-type distributions were statistically similar in all patient groups. Forty-one percent of patients were female and 59% were male. There was not a significant difference with regard to either gender (*F*_1,84_ = 0.13, *P* = .7213) or type of cleft (*F*_5,79_ = 0.69, *P* = .6305).

Forty-four percent of patients underwent straight-line repair with aggressive intravelar veloplasty (IVV), 36% underwent repair with the Furlow double z-plasty, and 20% underwent correction using the straight-line repair without IVV. There was a statistically significant difference (*F*_2,83_ = 5.36, *P* = .0065) between the 3 types of repair. The mean number of tubes placed was 0.6000 ± 0.1225, 0.8519 ± 0.1776, and 1.4737 ± 0.2785 for IVV, Furlow double z-plasty, and straight-line without IVV repair, respectively. The timing of tympanostomy tube placement trended toward statistical significance (*F*_2,83_ = 3.02, *P* = .0540). The estimated mean number of tubes was 1.4286 ± 0.4518, 0.6964 ± 0.1115, and 1.1304 ± 0.2217 when the initial set was placed before palatoplasty, initial placement at palatoplasty, and initial placement after palatoplasty, respectively.

## DISCUSSION

Despite its inherent limitations, this study suggests that palatal musculature reconstruction via intravelar veloplasty or reorientation via Furlow double z-plasty significantly reduced the total number tympanostomy tube sets in this population. These data are consistent with recent literature trend indicating improvement in Eustachian tube function and decrease in OME after palatoplasty with either IVV or a Furlow double z-plasty repair.^22^^-^26 Patients who underwent initial tympanostomy tube placement at the time of initial palatoplasty trended toward improved COME, likely constrained by a small sample size. Tympanostomy tube placement at the time of initial palatoplasty may be a viable addition to institutional protocol for cleft lip/palate patient base.

Even with meticulous repair, however, Eustachian tube dysfunction may be inherited in this population as a structural anatomic defect leaving OME to persist after palatal surgery.^10^^,^^12^^,^^22^^,^^27^^,^^28^ A recent study by Antonelli et al^13^ indicated no significant difference between the type of repair used and the eventual insertion of tympanostomy tubes. The discrepancy between findings is not apparently clear but may be due to the various limitations, surgical technique alterations, and sample size. Additional research with larger sample sizes may aid in noting the subtle differences in palatal and Eustachian tube function as a result of varying palatoplasty techniques. There is evidence, however, to support beliefs that the anatomic disruptions intrinsic to palatal clefting may cause irreversible tubal dysfunction or constriction that may not be amenable via conventional repairs alone.^12^^,^^27^ Alper et al^23^ also investigated the recovery of Eustachian tube function after palatal repair through the use of the double-opposing z-plasty. While the percentage of ears showing tubal dilation improved from approximately 27% to approximately 60%, a subpopulation of children with tubal dysfunction remains.^10^^,^^22^^,^^23^

## CONCLUSION

Despite its inherent limitations, this study suggests that palatal musculature reconstruction via intravelar veloplasty or reorientation via Furlow double z-plasty improves the resolution of COME in the population with cleft palate. Institutional protocols that include tympanostomy tube placement at the time of palatoplasty trended toward improved COME.
